# PP87 A Descriptive Study Of The Use Of Data Visualization In Full Health Technology Assessment Reports: 2021 To 2023

**DOI:** 10.1017/S0266462324002551

**Published:** 2025-01-07

**Authors:** Estefania Herrera Ramos, Rocío Rodríguez-López, Rebeca Isabel-Gómez, María Piedad Rosario Lozano, Beatriz Casal-Acción, Roland Pastells Peiró, Lorea Galnares-Cordero

## Abstract

**Introduction:**

Data visualization is a powerful communication tool to facilitate the understanding of a complex process or data. Data visualization has been used in health technology assessment (HTA) reports for a long period (e.g., PRISMA flow diagrams, critical assessments, or GRADE). This study aims to investigate the number and manners in which HTA reports have been using descriptive data visualization for the years 2021 to 2023.

**Methods:**

The international HTA database was used to identify and download the HTA reports from the years 2021 to 2023. We applied the database limits: full HTA and completed reports. The records were imported into a Microsoft Excel spreadsheet and screened by eight independent researchers applying the inclusion criteria: full HTA (according to methodological definition), access to the full text, and use of data visualization with a descriptive purpose (we excluded PRISMA flow diagrams, forest plots, and others). The data were observed with the software Power BI. Our analysis included variables such as agency name, country, section, type of visualization, and software.

**Results:**

From the international HTA database, 1,128 records were exported: 89 records were directly excluded from this set as they were tagged as ongoing or other types of reports in the database; 1,039 records were screened. Around 30 percent of records were included for the data visualization screening criteria after fulfilling our inclusion criteria (full HTA and available full text). Finally, 12 percent of the reports included data visualizations for a descriptive purpose of their results or conclusions. The countries with a higher number of records included in this analysis were Germany (28%), Canada (18%), the UK (16%), and Spain (11%).

**Conclusions:**

In our sample, we observed that data visualization is not widely used so far to describe outcomes and/or conclusions from HTA reports. An additional finding was the number of records tagged as full HTA in the international HTA database that were excluded as non-full HTA from our study.

